# Benefits of Femtosecond Laser 40 MHz Burst Mode for Li-Ion Battery Electrode Structuring

**DOI:** 10.3390/ma17040881

**Published:** 2024-02-14

**Authors:** Aurélien Sikora, Laura Gemini, Marc Faucon, Girolamo Mincuzzi

**Affiliations:** Alphanov, Aquitaine Institute of Optics, Rue F. Mitterrand, 33400 Talence, France

**Keywords:** battery, electrode, femtosecond laser, burst, micromachining

## Abstract

In Li-ion batteries, ion diffusion kinetics represent a limitation to combine high capacity and a fast charging rate. To bypass this, textured electrodes have been demonstrated to increase the active surface, decrease the material tortuosity and accelerate the electrolyte wetting. Amongst the structuring technologies, ultrashort pulse laser processing may represent the key option enabling, at the same time, high precision, negligible material deterioration and high throughput. Here, we report a study on the structuring of electrodes with both holes and grooves reaching the metallic collector. Electrochemical models emphasize the importance of hole and line dimensions for the performances of the cell. We demonstrate that we can control the hole and line width by adjusting the applied fluence and the repetition rate. In addition, results show that it is possible to drill 65 µm-deep and ~15 µm-wide holes in nearly 100 µs resulting in up to 10,000 holes/s. To further reduce the takt time, bursts of 40 MHz pulses were also investigated. We show that bursts can reduce the takt time by a factor that increases with the average power and the burst length. Moreover, at comparable fluence, we show that bursts can shorten the process more than theoretically expected.

## 1. Introduction

To increase autonomy and enhance the practicality of electric vehicles, batteries with a high capacity and fast charging rates are required [1]. A key parameter driving the optimization of these two properties is the electrode thickness. A thicker electrode will enhance the storage capacity of the battery while also slowing down the charging rate, since the path that the ions are required to travel will be longer. Therefore, to design the architecture of electrodes, a tradeoff has to be found between capacity and power. One way to overcome this issue consists of shortening the ion travel path by creating channels in the electrodes while maintaining an overall electrode thickness that can provide enough storage capacity [2,3,4]. Electrode structuring has been investigated using various techniques, such as freeze casting, phase separation and laser ablation [5]. All of these processes were shown to significantly enhance the charging rate capability [6]. Nevertheless, laser-based processes stand out based on their precision and their agnosticism to material and slurry. Additional benefits, such as lifespan extension [7,8], a reduction in lithium plating risk [9,10] and acceleration of the electrode wetting were demonstrated [9,11]. However, laser-based approaches suffer from the loss of matter and their relatively low throughput. To be considered for industrialization, the laser machining speed should match the pace of other manufacturing steps, such as electrode coating, drying, and cutting. Depending on the process and technology, this speed ranges from 25 to 300 m/min [12]. Currently, using a roll-to-roll machine [13], a line speed up to 5 m/s for drilling [14] and 10 m/s for grooving [15] were reported. Therefore, significant progress should be achieved to match the industrial requirements. Envisioned techniques for increasing the throughput consist of beam splitting for parallel processing and the utilization of high-average-power laser sources, with [16] or without a burst of MHz pulses [12]. For example, by using 21 beams, it was shown that the drilling time per processed area could be reduced by 88% [16]. In addition, using a burst composed of m pulses, the process rate can be, in principle, increased by a factor of m, and it has been reported that the removal efficiency can be improved as well, depending on the burst repetition rate, m and the material [17].

In addition to the process speed, one should also consider the pattern and the geometry of the structures. Electrode structuring can be performed by engraving lines, parallel [11,18] or cross hatched [19], or drilling holes (following a quadratic [20] or hexagonal pattern [21]). The impact of the pattern on the resulting performances is still ambiguous. Depending on the electrochemical modeling approach, it was shown, on the one hand, that the capability of these patterns is roughly similar [22] and, on the other hand, that hexagonal patterns are more efficient for fast charging [23]. In addition, these patterns do not present the same wetting properties and lead to different takt times [16,23]. For example, grooving parallel lines is less dependent on beam positioning precision than drilling. However, equidistant holes guarantee a homogeneous spreading of the electrolyte and, by punctually removing a small amount of matter, limits the risk of fragilization. Considering a hexagonal pattern, several modeling works have underlined the importance of the hole diameter and the hole-to-hole distance. To optimize the electrochemical performances, they should be adapted to the material porosity, the electrode thickness and the charging/discharging current and chosen to limit the volume of removed matter [24,25,26]. Therefore, control of the hole geometry is necessary to optimize the electrochemical performances. Using a picosecond laser and a graphite anode, it has been demonstrated that the aspect ratio can be tuned by varying the repetition rate, the applied fluence and the use of 5-pulse bursts at 82 MHz [16]. However, the burst-regime laser processing of lithium Nickel Manganese Cobalt oxides (NMC), one of the most used materials for cathodes in lithium-ion batteries [1], and the effect of the burst length have not been investigated. Here, we aim to address the two key issues in electrode laser structuring: the control of the structure geometry and the throughput increase. In the first part, we show how to control line and hole widths engraved in NMC and graphite-based electrodes using a femtosecond laser. In the second part, we show how to tailor the hole width in the NMC and reduce the process time by varying the length of a 40 MHz burst. As the benefits of laser structuring on electrochemical performances have already been abundantly reported [7,8,9,10,11,27], electrochemical characterizations will not be presented here. For example, by drilling graphite electrodes in a hexagonal way, Chen et al. could improve the capacity retention [7]. Meyer et al., through the line structuring of both electrodes, could increase the discharge capacity and the lifetime by a factor 3 [8]. Dunlap et al., through line structuring NMC and graphite electrodes, showed an improvement in charge cycling performance and a reduction in the Li-plating risk [9]. Habedank et al., by drilling graphite electrodes, showed a reduction in the impedance and a 27% enhancement in the discharge capacity retention [10]. Park et al. showed that femtosecond laser structuring allowed for a reduction in internal resistances and an increase in the specific capacity for current rates beyond 0.2C [27]. Rather, this study will focus on the capabilities and understanding of the laser structuring process using a burst mode approach with the objective of understanding possible improvements in the processing time and ablation quality.

## 2. Materials and Methods

**Figure 1:**
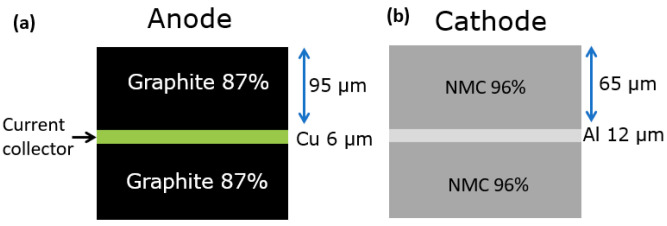
Schematized cross-section views of the graphite (**a**) and lithium Nickel Manganese Cobalt oxides (NMC) (**b**) electrodes.

Experiments were performed using two kinds of double-side coated electrode (see Figure 1). The first one, the anode, is composed of a 6 µm-thick copper current collector (CC) and a 95 µm-thick graphite-based coating (hard graphite 87%; PVDF 13% and dispersant). The second one, the cathode sample, is composed of a 12 µm-thick aluminium current collector and a 65 µm-thick NMC-based coating (LiNi_0.84_MnCoO_2_ 96%; C 1.8%, PVDF 1.8% and dispersant).

Two sets of experiments were performed in which the influence of the applied fluence on the geometry and the process time was investigated: the first set in a single-pulse regime and the second set in a 40 MHz-burst regime. For experiments in a single-pulse regime, the samples were processed with a laser (Tangerine HP by Amplitude, Pessac, France) delivering 350 fs pulses at a wavelength of 1030 nm. The beam was circularly polarized by means of a quarter waveplate, magnified 2.5 times with a telescope, injected in a galvanometric scanner (Excelliscan 14 by Scanlab, Puchheim, Germany) and focused with a 100 mm F-Theta lens. The resulting spot diameter in the focal plane was evaluated to be ~25 µm. Holes were machined by percussion drilling. Lines were engraved at scanning velocities of 0.8 m/s and 4.6 m/s at respective repetition rates (*f*) of 87 kHz and 1 MHz.

For experiments in a 40 MHz-burst regime, the samples were processed with a laser (Satsuma HP2 by Amplitude, Pessac, France) delivering 350 fs pulses at a wavelength of 1030 nm. The burst mode is clocked at 40 MHz and allows for a maximum of 32 successive pulses. Upon burst length variation, the average power is conserved, i.e., the energy is distributed between the pulses in the burst. The beam was circularly polarized by means of a quarter waveplate, magnified 2 times with a telescope, injected in a galvanometric scanner (hurrySCAN II 14 by Scanlab, Puchheim, Germany) and focused with a 100 mm F-Theta lens. The resulting spot diameter in the focal plane was ~27 µm. Hereafter, a burst composed of *m* pulses will be noted as B*m*, which will be also later referred to as the burst length. Holes were all drilled at f = 100 kHz with burst lengths varying between 1 (B1) and 32 pulses (B32). The applied fluence *F* was calculated considering the energy of one pulse in the burst.

In this study, all the measurements (hole and line width and process time) were performed once the ablation depth reached the surface of the CC, as required to optimize the electrochemical performances [25]. This protocol was adopted to facilitate the comparison between the processed samples with respect to the processing time at a constant ablation depth and to evaluate whether the laser structuring process could be performed without damaging the CC.

After laser machining, the samples were prepared for cross-section observation by embedding them in a resin made by mixing VariKleer (Buehler, Lake Bluff, IL, USA) 20-3592 liquid and 20-3591 powder. Then, the samples were polished with a grinder-polisher (EcoMet 300 by Buehler, Lake Bluff, IL, USA) using SiC abrasive paper (CarbiMet by Buehler, Lake Bluff, IL, USA) with a grit size 600 [P1200].

## 3. Results and Discussion

### 3.1. Electrode Structuring in Single-Pulse Regime

**Figure 2:**
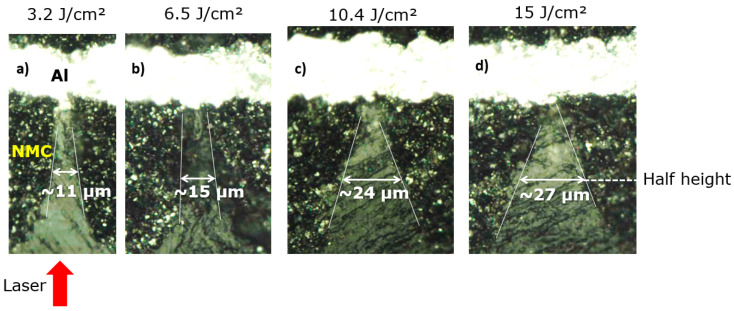
Cross-sections of drilled holes in an NMC electrode at various fluences, from 3.2 J/cm^2^ (**a**) to 15 J/cm^2^ (**d**), imaged via optical microscopy.

Figure 2 shows cross section views of holes obtained after drilling in the NMC at 87 kHz by applying various *F* values. For each *F*, the number of pulses was adjusted to reach the CC without damaging it. We can notice that by increasing *F* from 3.2 J/cm^2^ (a) to 15 J/cm^2^ (d)*,* the half-height width *w* of the hole increases from 11 to 27 µm.

**Figure 3:**
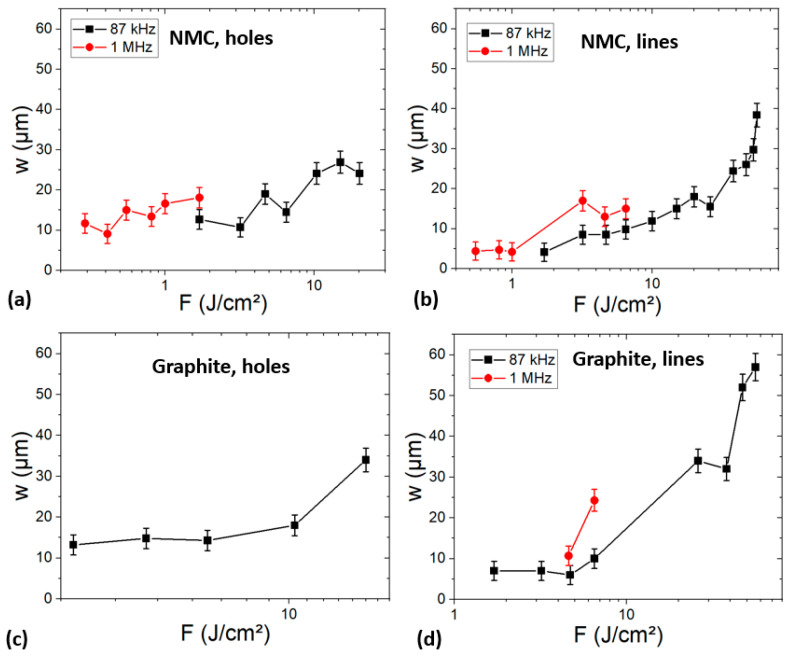
Half-height width evolution of holes or lines machined in NMC (**a**,**b**) and graphite (**c**,**d**) electrodes as a function of the applied fluence.

To increase the process throughput, the experiments were performed at 1 MHz for holes and lines on both graphite and NMC electrodes. The measured *w* values are plotted in the Figure 3. Firstly, in all cases, we observe an increase in *w* with *F*. This is consistent with theory as the diameter of an ablated crater increases logarithmically with *F* [28,29]. Secondly, for a given *F*, the measured *w* is larger at 1 MHz. Interestingly, for process time reduction, a given *w* can be obtained at a lower *F* at *f* = 1 MHz. This phenomenon can be attributed to heat accumulation, which allows for a reduction in the ablation fluence threshold [30]. Thus, *w* of holes or lines can be tailored to the desired dimensions, between a few µm and several tens of µm, by modifying *F* and *f*.

**Figure 4:**
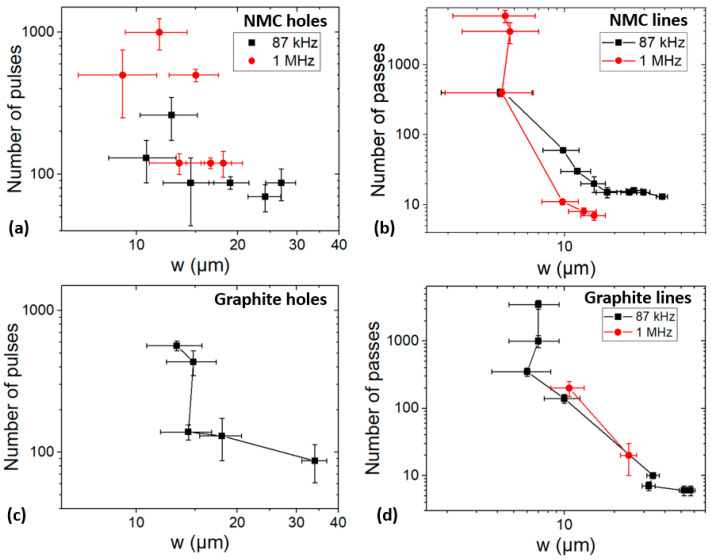
Evolution of the number of pulses or passes required to reach the current collector (CC) in NMC (**a**,**b**) and graphite (**c**,**d**) electrodes as a function of the half-height width.

To choose the best parameters allowing for a process time reduction with the desired geometry, it is helpful to know the required number of pulses or passes to reach the CC in the case of a hole or line of a defined *w*. Figure 4 presents the evolution of *w* as a function of the number of pulses and passes. We can observe, for both materials, that the required number of pulses and passes decreases of a factor of 10 as the hole or line width *w* doubles. This is caused by the higher fluence required for machining larger holes or lines. As the ablation rate increases with the fluence, fewer pulses or passes are necessary to reach the CC. Therefore, a trade-off must be found between the width and process time.

Increasing *f* is also beneficial for reducing the process time in the case of lines engraved in the NMC (Figure 4b). Beyond a half-height width of ~10 µm, fewer passes are necessary at 1 MHz. This phenomenon could be explained as well by heat accumulation making the ablation more efficient at 1 MHz. Additional data are required to conclude on the effect of *f* in the other cases (a, c, d).

### 3.2. Electrode Structuring in 40 MHz-Bursts Regime

#### 3.2.1. Effect of Number of Pulses Per Burst on Hole Geometry

**Figure 5:**
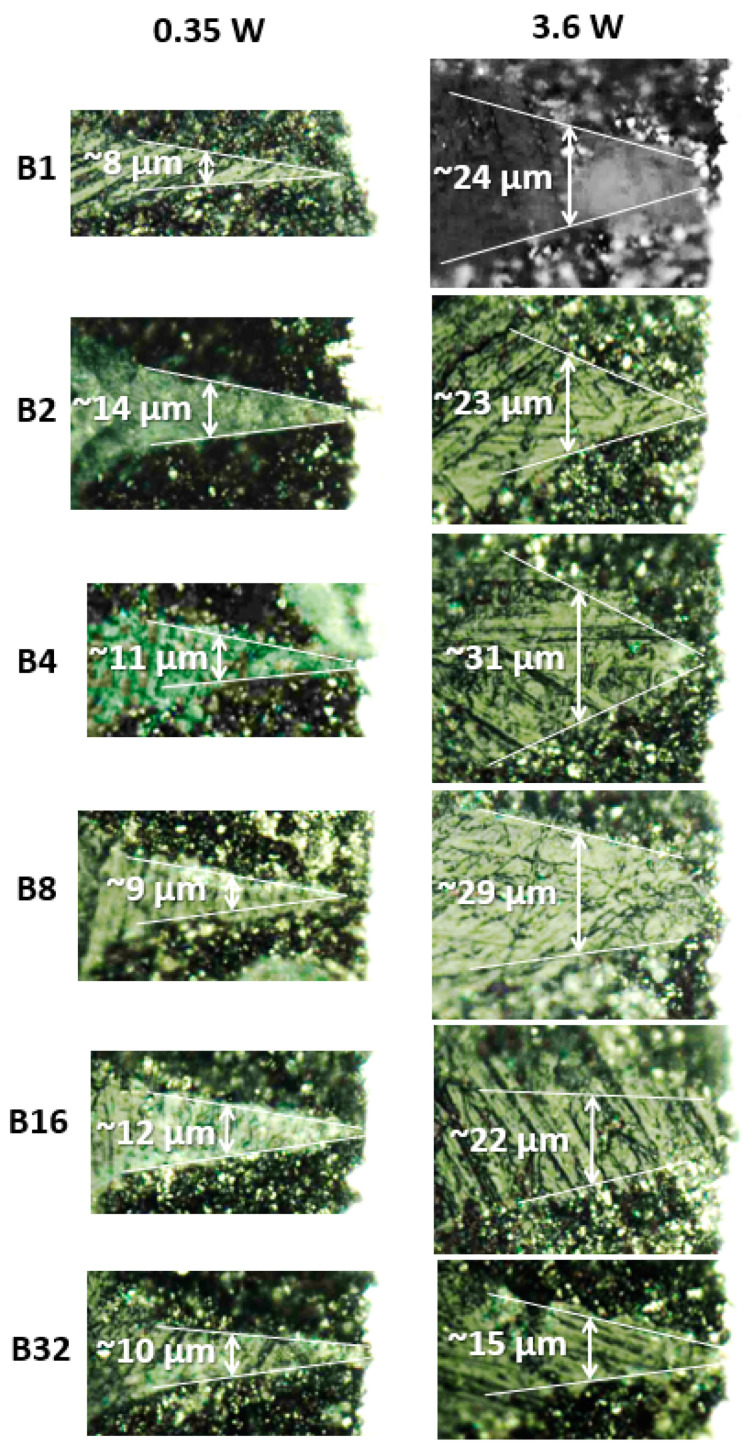
Cross-sections images of drilled holes in an NMC electrode using burst mode, from 1 pulse per burst (B1) to 32 pulses per burst (B32), at 0.35 and 3.6 W, imaged via optical microscopy.

NMC drilling has been tested in addition to utilizing a burst mode clocked at 40 MHz with *m* varying between 1 and 32 pulses (B1 to B32). Cross-section views of the holes drilled at an average power (*P*) of 0.35 W and 3.6 W for different burst lengths are presented in the Figure 5. First, no melting is observed regardless of the burst length and for both *P* values. At *P* = 0.35 W, the obtained *w* values are comparable independently from the burst lengths with an average value $\bar{w}\;$ of 10 µm and a standard deviation (SD) of 2 µm (relative SD = 20%). Similarly, at *P* = 3.6 W, *w* is not affected by *m*, although a larger $\bar{w}\;$ (24 µm) has been extracted, as expected by applying a larger fluence, with a relative SD: 21%.

**Figure 6:**
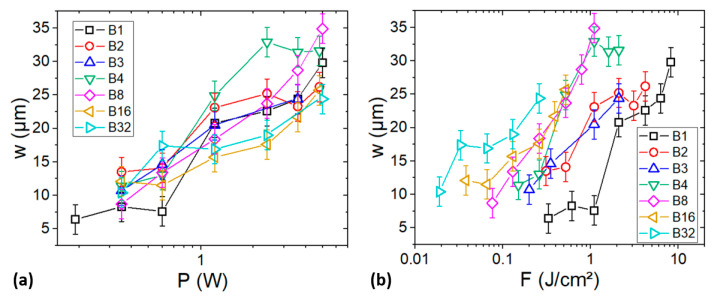
Evolution of the half-height width of holes drilled in the NMC (until collector) as a function of the average power (**a**) and the applied fluence (**b**) for different burst lengths.

The variation of *w* as a function of *P* for different burst lengths is presented in Figure 6a. If *m* is constant, the applied fluence increases with power and, therefore, induces an increase in *w*. This is confirmed by the experimental data shown in Figure 6a. Indeed, for each *m* value, *w* increases monotonically when *P* increases, except for B4 for which *w* stabilizes beyond 2.4 W.

Furthermore, for a given *P*, *F* decreases with the burst length. Thus, we expect a decrease in *w* with the burst length and a maximum for *m* = 1. However, we observe that the largest *w* is obtained with a burst length that depends on *P*. For example, at *P* = 0.3 W and *P* = 5 W, the widest holes are obtained, respectively, with B2 and B8.

For a further understanding, *w* has been plotted vs *F* as well (see Figure 6b). Firstly, as expected, *w* increases monotonically with *F*. Despite an identical hole depth (65 µm, i.e., the electrode thickness), *w* increases by factor up to 5 (between B8 and B1 at 1.1 J/cm^2^) by increasing the burst length. Secondly, regardless of the fluence, the narrowest and the widest holes are obtained, respectively, with B1 and B32. This behaviour can be attributed to a more important thermal accumulation induced at 40 MHz in the burst compared to 100 kHz for B1 [30]. At a low *F* (below ~0.3 J/cm^2^), *w* increases with *m*. However, for *F >* ~0.4 J/cm^2^, the achieved *w* values are similar for *m* ≥ 4.

#### 3.2.2. Ablation Model

To better understand the influence of the burst mode on the process speed, let us first consider a basic ablation model. The ablated depth per pulse in a single-pulse regime (*L_B_*_1_) can be approximated as

(1) $$L_{B1}=\alpha^{-1}ln\left(\beta P/F_{th}\right)$$

with *α* being the absorption coefficient, *β* a constant that depends on the beam waist size and *f*, and *F_th_* the ablation threshold fluence [29,31]. In a more general way, using the same total energy, i.e., at a constant *P* for any *m*, the ablated depth per burst *L_Bm_* is

(2) $$L_{Bm}\left(P\right)={m\alpha }^{-1}ln\left[\beta P/\left(mF_{th}\right)\right]$$

Then, in a first approximation, we can write the process time *t_Bm_*(*P*) as

(3) $$t_{Bm}\left(P\right)=\tau \alpha /\left\{fmln\left[\beta P/\left(mF_{th}\right)\right]\right\}$$

with *τ* being the NMC coating thickness.

**Figure 7:**
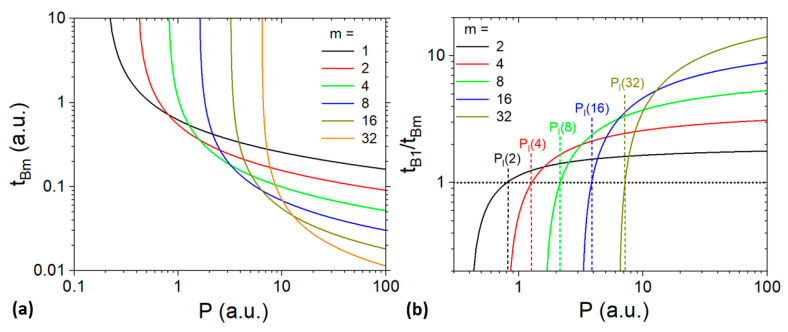
Modelized evolution of t_Bm_ (**a**) and of t_B1_/t_Bm_. (**b**) as a function of the average power for different burst lengths. Vertical dashed lines indicate the threshold power P_l_.

Equation (3) is plotted in Figure 7 using arbitrarily *β* = 1 m^2^/s and *F_th_* = 0.2 J/cm^2^. We observe that a threshold power *P_l_*(*m*), beyond which the process in the burst-regime becomes faster than in a single-pulse regime, exists and increases with *m*. Moreover, at a sufficiently high *P*, the shortest process time is obtained using the longest burst (B32). *P_l_*(*m*) can be calculated by equalizing *L_B_*_1_ and *L_Bm_*. Then, the following expression is obtained:

(4) $$P_{l}\left(m\right)=\beta F_{th}m^{m/\left(m-1\right)}$$

with *β* = π*ω*_0_^2^*f* and *ω*_0_ being the beam waist radius. *P_l_*(*m*) is a growing function, which is consistent with the observed increase in *P_l_*(*m*) with *m* (see Figure 7).

To better compare the effect of the burst length on the process time, it is more practical to consider *t_B_*_1_/*t_Bm_* (see Figure 7b). In this case, a ratio below 1 means that the process with a burst is longer than for a single-pulse regime (B1). The observed trends confirm that the process time reduction increases with both *P* and *m*. Furthermore, the process becomes faster than B1 if *P* is larger than *P_l_*(*m*) (vertical dashed lines). Using a basic modelling approach, bursts are shown to accelerate the process significantly beyond a threshold *P_l_*, which increases with *m*.

#### 3.2.3. Evolution of Processing Time with Number of Pulses per Bursts

**Figure 8:**
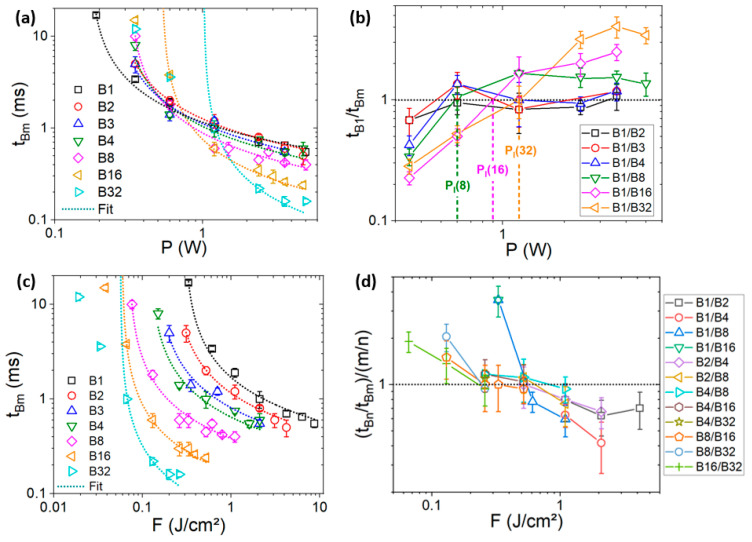
Evolution of the drilling time as a function of the average power (**a**) and the applied fluence (**c**). (**b**) Evolution of the ratio between the single pulse (B1) and the burst (Bm) process times as a function of the average power. Vertical dashed lines denote the threshold power P_l_. (**d**) Evolution of the ratio between the measured process time ratio and the expected ratio considering the number of pulses in the bursts.

The variation of *t_Bm_* with *P* is presented in the Figure 8a. For any *m*, *t_Bm_* decreases with *P*, as expected. Interestingly, beyond a threshold (around 1 W) depending on *m*, *t_Bm_* value with *m* ≤ 4 are comparable and when *P* > ~2 W, *t_Bm_* decreases by increasing *m*, in accordance with the model. Below this threshold, *t_Bm_* is the longest for the longest bursts. In this case, the removal rate is limited by *F*, which is lower for longer bursts. The data were fitted using Equation (3) (dotted lines in Figure 8a). There is a good agreement between the model and the data except at a low *P* for *m* = 16 and *m* = 32, for which the process times are shorter than expected. This discrepancy can be attributed to the increase in the ablation rate induced by thermal accumulation, which is more important for longer bursts.

To better evaluate the process time variation using bursts, the ratio between *t_B_*_1_ and *t_Bm_* was plotted as a function of *P* (see Figure 8b). We can observe that the ratio increases with *P* and *P_l_* (vertical dashed lines) increases with *m*, as expected from the model*,* varying from ~0.6 W for m = 8 to ~1.2 W for m = 32. The largest time decrease (factor ~4) is obtained for B32 at 3.6 W.

For a further understanding, *t_Bm_* was plotted vs. *F* (Figure 8c). As expected, for any *m*, *t_Bm_* decreases with *F*. Moreover, for any *F*, the larger the *m*, the shorter the *t_Bm_*. This could be attributed to the temporal distribution of the pulses using bursts. To have a better understanding, (*t_Bn_*/*t_Bm_*)/(*m*/*n*) (with *m* > *n*) was plotted vs *F* (Figure 8d). In this case, ratios larger than 1 correspond to conditions where the process time is shorter than expected considering only the number of pulses (*n* and *m*) in the bursts. It can be observed that the ratio decreases with *F*. Moreover, beyond a threshold of around 0.4 J/cm^2^, *t_Bm_* becomes longer than expected for any *m*. The largest improvement is observed for B16 (blue triangle symbol) at 0.33 J/cm^2^ for which the process time has been reduced ~3.5 times more than expected. However, B16 is not necessarily the optimal burst length. Indeed, as B1 and B32 were investigated in non-overlapped fluence ranges (see Figure 8c), the data for *t_B_*_1_*/t_B_*_32_ were not available. Thus, *F* and *m* appear to be crucial parameters for determining the efficiency of the burst mode, in agreement with other reports [17,32].

According to the model, if *α* and *F_th_* were constant, (*t_Bn_*/*t_Bm_*)/(*m*/*n*) should be always equal to 1 regardless of *F*, *n* and *m*. The observed variation (see Figure 8d) suggests that they are not. These parameters can be accessed by fitting the data presented in Figure 8c using the following equation:

(5) $$t_{Bm}\left(F\right)=\tau \alpha /\left[fmln\left(F/F_{th}\right)\right]$$

**Figure 9:**
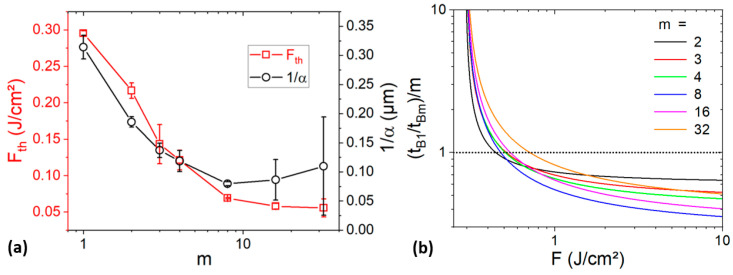
(**a**) Variation in *F_th_* and penetration depth 1/*α*, extracted from fits, as a function of *m*. (**b**) Variation in the ratio between the measured and expected process time considering *m*. *α* and *F_th_* extracted from the fits presented in Figure 8b were used.

Similarly to the evolution of *t_Bm_* with *P*, there is a good agreement between the experimental data and the model, except at a low *F* for the longest bursts (*m* = 16 and *m* =32). In these cases, heat accumulation is more important. As the model is based on optical absorption and does not consider thermal effects [31], discrepancy can occur. By fitting the data, it is possible to extract *α*(*m*) and *F_th_*(*m*). The evolution of these values is presented in the Figure 9a. We observe a large decrease (reduction by a factor ~4 between *m* = 1 and *m* = 8) and, beyond *m* = 8, a stabilization of both *F_th_* and *α* with *m*. The decrease in *F_th_* is expected considering the increase in thermal accumulation with *m* [30]. This could explain why the process with bursts is faster than expected at a given fluence (see Figure 8d). However, the process time is also proportional to the absorption coefficient, which increases with *m* as well. Thus, as 1/*α* and *F_th_* decrease simultaneously and have opposite effects, it is difficult to predict the evolution of the process time with the fluence. Nevertheless, it can be determined analytically by using equation 5 and the extracted parameters from the fits. To check that, (*t_B_*_1_/*t_Bm_*)/*m* was plotted versus *F* using equation 5 and the extracted *α*(*m*) and *F_th_*(*m*) values. We observe that the process time is shorter than expected if the applied fluence is sufficiently low (between 0.45 and 0.71 J/cm^2^, depending on *m*), in agreement with the experimental data (see Figure 8d). Thus, the process time with the burst can be shorter than expected because of the burst-induced variation in *F_th_* and *α*.

### 3.3. Comparison of Laser Processing Techniques

Table 1 presents a comparison of the processing time between results obtained in this work and the existing literature. The use of a burst-regime allows for a reduction in the processing time up to a factor of 4 with respect to a single-pulse regime (as shown in Figure 8) and by a factor > 4 if compared to results found in existing references [10,33,34]. Results presented in this work demonstrate that the process can become more efficient by varying the temporal and energetic distribution of the laser pulses. This outcome could be attributed to the different thermal dynamics in the burst-mode regime and single-pulse regime [30,35]. The more important thermal accumulation observed in the burst-mode regime allows for working at lower applied fluences by increasing the number of pulses at a constant average power. This way, it is possible to ablate closer to the optimal removal efficiency [29]. Ideally, the process could be shortened even further by increasing the average power through a multi-beam parallelization approach or by using a longer burst, as it is possible to deduce from Figure 7. Femtosecond laser sources, with up to hundreds of W average power, are currently industrially available. Considering a source able to deliver 100 W, the process time could be reduced by a factor of about 20 in the best-case scenario with respect to the results presented in this work, where only 5 W average power was employed.

The burst-mode regime allows for better control of the ablation topography as well. The aspect ratio of the ablated regions can be controlled by simply varying the burst length at a constant average power, as shown in Figure 5 and Figure 6.

## 4. Conclusions

In this study, the effects of the fluence and repetition rate on the geometry of engraved lines and drilled holes in NMC and graphite-based electrodes were investigated. We show that the half-height width increases with the applied fluence and the repetition rate. Drilling at 1 MHz allows for a reduction in the pulse energy required to reach a given width. Furthermore, it was demonstrated that the process time required to reach the collector decreases with the width, as the latter is directly dependant on the applied fluence.

Moreover, the effect of the burst length on the hole-drilling process has been investigated. Burst length was shown to affect the hole width and drilling time as well. At a comparable average power, bursts allow for a reduction in the process duration by a factor as high as ~4. The time reduction factor globally increases with the power and burst length. In addition, at a given fluence, it is shown that the process time can be shorter than expected considering only the number of pulses in the burst. This phenomenon, attributed to a burst-induced variation in the material properties, happens only if the applied fluence is below a limit, which depends on the burst length. Otherwise, the process time is reduced only because of the temporal distribution of the pulses. However, in specific conditions, bursts can be useless or detrimental to the process time. For example, no significant effects were found for burst lengths below 4 pulses. Moreover, below a certain threshold power, which depends on the burst length and the ablation fluence threshold, the process time increases with the burst length. Therefore, the burst can be beneficial only in a specific process window, which depends on the material properties. Moreover, the improvement in electrochemical performances must be checked in future work. With the right choice of parameters, by significantly accelerating the process, the implementation of the burst mode in the laser structuring of electrodes appears to be a promising method to significantly reduce the manufacturing costs.

## Figures and Tables

**Table 1:** Process parameters and corresponding minimum drilling time.

Pulse Duration (ps)	Average Power (W)	Repetition Rate (kHz)	Pulse Energy (µJ)	Hole Depth (µm)	Minimum Drilling Time (ms)	References
150	15	1200	12.5	43	0.7	[33]
150	3	1200	2.5	63	0.45	[10]
150	10	1200	8.3	71	0.6	[34]
0.35	5	100 (B32)	1.6	65	0.16	This work
0.35	5	100 (B1)	50	65	0.55	This work

## Data Availability

No new data were created or analyzed in this study. Data sharing is not applicable to this article.
